# Evaluating Dry Eye Disease Subtypes Based on Whole-Area Lipid Layer Thickness Assessment

**DOI:** 10.3390/jcm15093553

**Published:** 2026-05-06

**Authors:** Hyunmin Ahn, Young Joon Choi, Ikhyun Jun, Tae-im Kim, Kyoung Yul Seo

**Affiliations:** 1 Clear Eye Clinic, Pyeongtaek-si 18029, Gyeonggi-do, Republic of Korea; overhyun31@gmail.com; 2 Department of Medicine, Yonsei University Graduate School, Seoul 03722, Republic of Korea; 3 Department of Ophthalmology, Ajou University College of Medicine, Suwon-si 16499, Gyeonggi-do, Republic of Korea; singtomorrow@gmail.com; 4 Department of Ophthalmology, Yonsei University College of Medicine, 50-1 Yonsei-ro, Seodaemun-gu, Seoul 03722, Republic of Korea; hadesdual@yuhs.ac (I.J.); tikim@yuhs.ac (T.-i.K.)

**Keywords:** dry eye disease, meibomian gland dysfunction, tear film-oriented diagnosis, tear interferometry

## Abstract

**Background/Objectives:** Lipid layer thickness (LLT) is widely used to assess tear film status in dry eye disease (DED), but single-point measurements may not adequately reflect spatial lipid distribution driven by tear film dynamics. This study evaluated whether the combined assessment of inferior and superior corneal LLT provides additional clinical relevance for interpreting DED subtypes. **Methods:** This cross-sectional study included 614 eyes of 614 patients with DED. Inferior corneal LLT (LLT*_inf_*) was measured using a tear interferometer, and superior corneal LLT (LLT*_sup_*) was graded using an LED-based slit-lamp assessment. DED parameters, including meibomian gland expressibility (MGE), meibum quality, tear meniscus height, Schirmer I test, and fluorescein tear break-up patterns, were analyzed. **Results:** Low LLT*_inf_* showed worse meibomian gland function, with higher MGE scores than in the high LLT*_inf_* group (1.9 ± 0.8 vs. 1.2 ± 0.9, *p* < 0.001). Higher LLT*_inf_* was associated with lower aqueous parameters, and aqueous deficiency was observed in 57.4% of the high LLT*_inf_* group increasing to 83.0% when LLT*_sup_* was low. Combined LLT*_inf_*–LLT*_sup_* assessment improved the prediction of aqueous deficiency compared with LLT*_inf_* alone (AUC, 0.719 vs. 0.559). Improvement for moderate-to-severe MGD was smaller (AUC 0.731 vs. 0.653). **Conclusions:** LLT reflects not only lipid secretion, but also aqueous-driven distribution. The combined assessment of LLT*_inf_* and LLT*_sup_* may improve the interpretation of LLT findings and provide additional insight into tear film dynamics in DED. However, its predictive performance remains moderate, suggesting that this approach is considered a complementary interpretive framework rather than a standalone diagnostic tool.

## 1. Introduction

Dry eye disease (DED) is a multifactorial disorder defined by the loss of homeostasis of the tear film and ocular surface microenvironment [1]. This imbalance triggers a ‘vicious cycle’ where tear film instability and hyperosmolarity drive inflammatory signaling pathways and ocular surface damage [2]. Traditionally, DED has been classified into aqueous-deficient and evaporative subtypes, a framework later expanded by the Asian Dry Eye Society to include mucin dysfunction [3]. However, the recent TFOS DEWS III report emphasizes that such broad categorization often fails to capture the multiple, coexisting pathogenic drivers unique to each patient.

The component-based perspective emphasizes the evaluation of individual tear film components alongside conventional DED assessments. However, these approaches are largely based on static measurements and may not fully capture tear film behavior. Recent efforts have therefore focused on dynamic aspects of the tear film, including its distribution and stability across the ocular surface. Representative methods include fluorescein tear break-up pattern (FTBUP) analysis and tear interferometric lipid layer thickness (LLT) assessment [4,5].

LLT is commonly used as an indicator of tear film lipid status [6]. However, its interpretation is complex because LLT is influenced not only by meibum secretion, but also by tear film dynamics, including blinking and aqueous flow [7,8,9]. As a result, increased LLT does not necessarily indicate increased lipid secretion. In particular, aqueous deficiency may impair the upward spread of the lipid layer, leading to lipid accumulation in the inferior cornea despite reduced effective distribution across the ocular surface [8,9]. Thus, an increased inferior LLT does not necessarily indicate increased lipid expression, but may instead reflect impaired lipid distribution.

Despite these limitations, most clinical studies have relied on interferometric LLT measurements obtained from a limited corneal region, typically the inferior or central cornea [10,11,12]. This single-point approach lacks spatial information and may lead to misinterpretation of LLT, as it cannot distinguish between lipid accumulation and impaired lipid distribution.

Accordingly, evaluating LLT across different corneal regions may provide additional insight into tear film dynamics. We hypothesized that combined assessment of inferior and superior corneal LLT would allow differentiation between lipid secretion and lipid distribution patterns. This study aimed to determine whether integrating inferior and superior LLT improves the interpretation of LLT by distinguishing lipid accumulation from impaired distribution in DED subtypes.

## 2. Methods

### 2.1. Study Design

This cross-sectional study conducted at Severance Hospital, Yonsei University College of Medicine, and enrolled consecutive DED patients from January 2021 to December 2024. The study protocol was approved by the Institutional Review Board of Severance Hospital (IRB approval number: 2024-3494-001) and adhered to the tenets of the Declaration of Helsinki.

### 2.2. Subjects

The study subjects were DED patients with following criteria. The inclusion criteria were (1) age ≥ 20, (2) Ocular Surface Disease Index (OSDI) ≥ 13 scores (mild and/or over), and (3) fluorescence tear break-up time (TBUT) ≤ 10 s [13]. The exclusion criteria were history of ocular trauma or surgery within 6 months, history of contact lens within 3 months, and history of dry eye treatments except preservative-free artificial tear within 3 months.

### 2.3. Sample Size

The sample size calculation was based on a previous study that analyzed dry eye subtypes and LLT of the inferior cornea [14]. The mean LLT in the inferior cornea was reported as follows: (1) aqueous-deficient subtype: 97 ± 6 nm (*n* = 11), (2) evaporative subtype: 72 ± 25 nm (*n* = 142), (3) mixed subtype: 71 ± 27 nm (*n* = 144), and (4) unclassified dry eye: 74 ± 11 nm (*n* = 12). These values were used as the basis for sample size estimation. The effect size (Cohen’s f) was calculated as 0.19, using the weighted mean and weighted variance based on the number of subjects in each group. Since normality assumptions might be violated, the effect size was adjusted using the Rank–Biserial Correlation from the Kruskal–Wallis test. To approximate an equivalent effect size, Cohen’s f was converted by dividing by 0.8, yielding an adjusted f of 0.24. With a significance level (α) of 0.05 and power (1 − β) of 0.95, the estimated required sample size was 307 subjects. In order to account for LLT measurements from both the inferior and superior cornea, the required number was conservatively doubled, resulting in a final estimated sample size of 614 participants.

### 2.4. Tear Interferometry and Lipid Layer Thickness

Tear interferometry was performed using two methods: a non-invasive, commercially available interferometer (TearScience™ LipiView^®^ II Ocular Surface Interferometer, Johnson & Johnson Vision, Jacksonville, FL, USA) for the inferior cornea and a manual LED plate for the superior cornea. Both techniques utilize specular reflection of white light from the TFLL to assess its color and uniformity.

LLT obtained from the tear interferometer represents the LLT in the inferior cornea and the average LLT value is defined as inferior corneal LLT (LLT*_inf_*). The average value was used for individual measurements. When the number of measurements exceeded two, both upper and lower extreme values were excluded from the calculations. Patients with highly discontinuous or irregular LLT patterns due to poor cooperation, as well as those with a C-factor below 0.90, an indicator of measurement reliability in the graphical summary, were excluded. The detailed methodology followed that of a previously published study [9]. LLT*_inf_* was classified into three grades using clinically distinguishable cutoff values of tear interferometric patterns identified from a Gaussian mixture model considering previous research (Supplementary Materials S1): (1) grade L (LLT*_inf_* < 55.1 nm), (2) grade M (55.1 nm ≤ LLT*_inf_* < 89.5 nm), and (3) grade H (89.5 nm ≤ LLT*_inf_*) [6,15].

Given the current lack of automated quantitative tools for the superior cornea, superior corneal LLT (LLT*_sup_*) was measured with a whitish LED plate under slit-lamp examination, followed by anterior segment photography [16]. The superior cornea was illuminated with the LED plate and classified into three grades using a modified version of a previously established classification system (Supplementary Materials S2): (1) grade L (dark, uniform distribution), (2) grade M (gray, uniform or non-uniform distribution), and (3) grade H (colored, non-uniform distribution) [17]. The intra-/interclass correlation coefficient (ICC) of LLT*_sup_* among three independent examiners was 0.791 [95% confidence interval (CI) 0.642–0.941] (Supplementary Materials S3).

The LLT group was categorized into nine (3 × 3) subgroups based on the LLT grades of LLT*_inf_* and LLT*_sup_*. Each group was labeled using the format Group*_inf-sup_*, where “inf” represents the LLT*_inf_* grade and “sup” represents the LLT*_sup_* grade. For example, if LLT*_inf_* was grade H and LLT*_sup_* was grade M, the group was labeled as Group*_H-M_*.

### 2.5. Dry Eye Disease Assessments

Conventional DED assessments included OSDI [0–100 scores], TBUT, non-invasive keratographic tear break-up time (NIKBUT) measured using the Keratograph^®^ 5 M (Oculus, Wetzlar, Germany), and corneal staining score (CSS, [0–3 grades]) based on the ocular surface staining score of the Sjögren’s International Collaborative Clinical Alliance (SICCA) [18,19,20,21].

Tear volume was measured with the Schirmer I test performed for 5 min without anesthesia and tear meniscus height (TMH) at the central tear meniscus area using Keratograph^®^ 5 M [22]. Aqueous deficiency was defined as Schirmer I test ≤ 5 mm or TMH < 200 μm [1].

Meibomian gland functionality was evaluated in the central eight glands of the upper and lower eyelids based on the guidelines of the 2011 International Workshop on MGD [23]. Meibomian gland expression (MGE) was measured at a pressure of 0.3 pounds per square inch (psi; approximately 15 mmHg) using a commercially available instrument, the Meibomian Gland Evaluator (Johnson & Johnson Vision, Jacksonville, FL, USA; Supplementary Materials S4), to ensure standardized and equivalent pressure application [24]. MGE was categorized into four grades: (1) grade 0 (all glands expressible, none), (2) grade 1 (50% and over of glands expressible, minimal to mild), (3) grade 2 (less than 50% of glands expressible, moderate), and (4) grade 3 (no glands expressible, severe). Meibum quality (MQ) was assessed as the average value of meibum feature scores: (1) grade 0 (clear fluid), (2) grade 1 (cloudy fluid), (3) grade 2 (cloudy particulate fluid), and (4) grade 3 (toothpaste-like secretion or no secretion). The following features were assessed for lid margin abnormalities: (1) meibomian gland plugging, (2) telangiectasia, (3) anterior shift of mucocutaneous junction (MCJ), (4) notching, and (5) lid margin desquamation. MGD grade was assigned according to the most severe finding among MGE, MQ, and lid margin abnormalities.

FTBUPs were evaluated using slit-lamp examination videoclips. Five distinct patterns of fluorescein tear break-up have been described: spot break (SB), dimple break (DB), area break (AB), line break (LB), and random break (RB). According to the ADES guidelines, SB and DB patterns are indicative of decreased wettability dry eye, RB suggests evaporative dry eye, and AB or LB are associated with aqueous-deficient dry eye [4]. Three independent examiners assessed the videoclip, and the ICC was 0.772 [95% CI 0.666–0.876] (Supplementary Materials S2).

### 2.6. Statistical Analysis

Statistical analyses were conducted using Python version 3.18. Since LLT*_sup_* is influenced by the upward movement of the aqueous layer and reflects lipid distribution from the inferior to the superior cornea, a stratified analysis of LLT*_sup_* within each LLT*_inf_* group was conducted. For the comparison of LLT groups, nonparametric methods were chosen due to the violation of normality and homogeneity of variance assumptions. The Kruskal–Wallis test was used for continuous variables, the Jonckheere–Terpstra test was applied for ranked variables, and the Chi-square test was used for nominal variables. Bonferroni’s test was conducted as a post hoc analysis following the Kruskal–Wallis test. For the correlation analysis of variables, Spearman’s rho correlation test was conducted. To analyze the relative availability of concomitant measurement of inferior and superior corneal LLT, Receiver Operating Characteristic (ROC) curve analysis was performed, and the area under the curve (AUC) was calculated by logistic regression. The performance of LLT*_inf_* alone was compared to a model LLT*_sup_* and a model incorporating both LLT*_inf_* and LLT*_sup_* in (1) distinguishing MGE grade 0 from grades 1, 2, and 3, (2) distinguishing MGE grades 0 and 1 from grades 2 and 3, and (3) predicting aqueous deficiency. Both in previous studies, MGE showed a higher correlation with LLT*_inf_* compared to MQ [25]. Moreover, since MGE and MQ exhibited high collinearity, MGE was selected to evaluate the performance in meibomian gland functionality. A significant level of *p*-value < 0.05 was considered statistically significant, and all statistical tests were performed at a 95% confidence level.

## 3. Results

### 3.1. Characteristics of Subjects

A total of 614 eyes from 614 patients were included in this study. No subjects were assigned to Group*_L-H(inf-sup)_*, and analyses were conducted on the other eight groups. The distribution of subjects across these groups is presented in Supplementary Materials S5. The mean age of study subjects was 49.5 ± 15.4 years, and 77.9% were female. The mean OSDI score was 28.7 ± 15.6, TBUT was 3.5 ± 2.5 s, and CSS was 0.5 ± 1.4 as detailed in Table 1 and Supplementary Materials S6.

### 3.2. Inferior Corneal Lipid Layer Thickness and Dry Eye Assessments (Table 2)

Regarding meibomian gland functionality, statistically significant correlations were observed between LLT*_inf_* and MGE (*r* = −0.266, *p* < 0.001). Specifically, patients in the low LLT*_inf_* group exhibited significantly worse MGE (1.9 ± 0.8) compared to those in the grade M (1.1 ± 0.9) and grade H (1.2 ± 0.9) groups (both *p* < 0.001). MQ followed a similar trend, showing the most severe degradation in the low LLT*_inf_* group (2.5 ± 0.8) compared to other grades (*p* < 0.001). These findings suggest that a thin lipid layer at the inferior cornea is a indicator of reduced meibum delivery and poor meibum quality (see also Supplementary Materials S7).

**Table 2:** Comparison of Key Dry Eye Parameters According to Inferior Corneal LLT (LLT*_inf_*) grades.

	LLT*_inf_* Grade	Value	*p*-Value
MGE (grades)	Low	1.9 ± 0.8	<0.001 (vs. M) *
	Middle	1.1 ± 0.9	1.000 (vs. H)
	High	1.2 ± 0.9	<0.001 (vs. L) *
MQ (grades)	Low	2.5 ± 0.8	<0.001 (vs. M) *
	Middle	2.1 ± 0.9	1.000 (vs. H)
	High	2.0 ± 0.9	<0.001 (vs. L) *
TMH (μm)	Low	219.7 ± 39.3	0.005 (vs. M) *
	Middle	207.0 ± 48.9	1.000 (vs. H)
	High	200.4 ± 41.8	0.001 (vs. L) *
Schirmer (mm)	Low	7.3 ± 4.9	0.467 (vs. M)
	Middle	6.7 ± 3.9	0.373 (vs. H)
	High	6.3 ± 3.6	0.231 (vs. L)
AD (%)	Low	42.1	0.158 (vs. M)
	Middle	52.3	0.807 (vs. H)
	High	57.4	0.015 (vs. L) *
TBUPs ^†^ (%)	Low	8.6/30.7/47.9/12.1/0.7	<0.001 (vs. M) *
	Middle	19.4/20.9/35.3/23.6/0.8	<0.001 (vs. H) *
	High	10.6/34.3/23.2/28.3/4.6	<0.001 (vs. L) *

Abbreviation: AD, aqueous deficiency; LLT, lipid layer thickness; MGE, meibomian gland expressibility; MQ, meibum quality; TBUP, tear break-up pattern; TMH, tear meniscus height. * *p* < 0.05, *p*-value was calculated with Kruskal-Wallis test for continuous variables, Jonckheere-Terpstra test for ranked variables, and chi-square test for nominal variables. Bonferroni’s test was conducted as a post-hoc analysis following Kruskal-Wallis test. ^†^ Tear break-up patterns are listed in the order of dimple break (DB), spot break (SB), random break (RB), line break (LB), and area break (AB). Each value represents the percentage of cases exhibiting each pattern.

In contrast, higher LLT*_inf_* was associated with decreased aqueous parameters and a higher prevalence of aqueous deficiency. TMH significantly decreased as LLT*_inf_* increased, with the lowest values observed in grade H (200.4 ± 41.8 μm) compared to grade L (219.7 ± 39.3 μm, *p* = 0.001). Correspondingly, the prevalence of aqueous deficiency rose from 42.1% in the low LLT*_inf_* group to 57.4% in the high LLT*_inf_* group. While Schirmer I test values showed a decreasing trend with higher LLT*_inf_* (7.3 mm in grade L vs. 6.3 mm in grade H), these differences did not reach statistical significance.

Significant associations were also found between LLT*_inf_* and FTBUPs (*p* < 0.001). RB were the most predominant pattern in the low LLT*_inf_* group (47.9%), whereas SB and LB were more prominent in the high LLT*_inf_* group, accounting for 34.3% and 28.3% of cases, respectively. Interestingly, no statistically significant difference was observed in subjective symptom severity (OSDI) among the different LLT inf grades (*p* = 0.326), suggesting that regional lipid layer thickness may not directly reflect overall symptom burden.

### 3.3. Subgroup Analysis of Superior Corneal Lipid Layer Thickness (Figure 1)

Stratified analysis revealed that LLT*_sup_* grades provide critical diagnostic context for interpreting inferior corneal measurements (see also Supplementary Materials S7). Within the high LLT*_inf_* group, statistically significant differences were observed in subjective symptoms and clinical signs based on LLT*_sup_* distribution. For instance, patients in Group*_H-L_* exhibited significantly worse OSDI scores (32.2 ± 9.8) and higher corneal staining scores (1.1 ± 1.3) compared to those in Group*_H-H_* (both *p* < 0.05). In contrast, TBUT was shortest in Group*_H-H_* (2.4 ± 1.4 s) compared to other subgroups within the high LLT*_inf_* category (*p* < 0.001).

**Figure 1:**
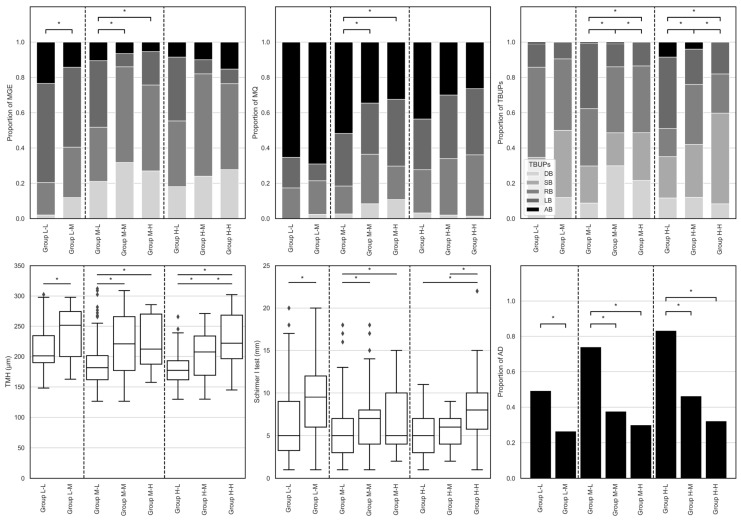
**Meibomian gland functionality (top left and middle), tear break-up patterns (TBUPs, top right), and aqueous volume (bottom) of lipid layer thickness groups.** Group labels are shown as LLT*_inf_*–LLT*_sup_* grades: the first letter indicates the inferior lipid layer thickness (LLT*_inf_*) grade, and the second letter indicates the superior lipid layer thickness (LLT*_sup_*) grade. Accordingly, the subgroup blocks separated by dashed vertical lines represent LLT*_inf_* grade L (L-L and L-M), LLT*_inf_* grade M (M-L, M-M, and M-H), and LLT*_inf_* grade H (H-L, H-M, and H-H), respectively. Meibomian gland functionality was evaluated using meibomian gland expressibility (MGE) and meibum quality (MQ). Aqueous volume was assessed based on tear meniscus height (TMH, bottom left) measured by Keratograph 5 M^®^ and Schirmer I test (bottom middle). Aqueous deficiency (AD, bottom right) was defined as either TMH < 200 μm or Schirmer I ≤ 5 mm. * *p* < 0.05.

The most explicit relationship was found between LLT*_sup_* and aqueous volume parameters across all LLT*_inf_* grades. Both TMH and Schirmer I test values increased consistently as the LLT*_sup_* grade increased (all *p* < 0.001). Notably, the prevalence of aqueous deficiency was remarkably higher in groups with low superior distribution, reaching 83.0% in Group*_H-L_* compared to approximately 30% in groups with high superior distribution.

Significant shifts in FTBUPs were also identified in relation to superior lipid distribution. The proportion of SB increased significantly as LLT*_sup_* increased in the high LLT*_inf_* group, while the proportion of LB increased as LLT*_sup_* decreased (all *p* < 0.001).

### 3.4. Performance of Combined LLT_inf_ and LLT_sup_ Assessment (Figure 2)

The results confirm that the combined assessment of LLT*_inf_* and LLT*_sup_* yields a significantly higher performance in identifying DED subtypes compared to LLT*_inf_* assessment alone. Specifically, the prediction for aqueous deficiency showed the most substantial improvement, with the AUC increasing from 0.56 (LLT*_inf_* grade) to 0.72 for the combined assessment. In contrast, the additional diagnostic value of LLT*_sup_* for MGE was less pronounced than that for aqueous deficiency, with the AUC for moderate-to-severe MGE increasing from 0.65 to 0.73.

**Figure 2:**
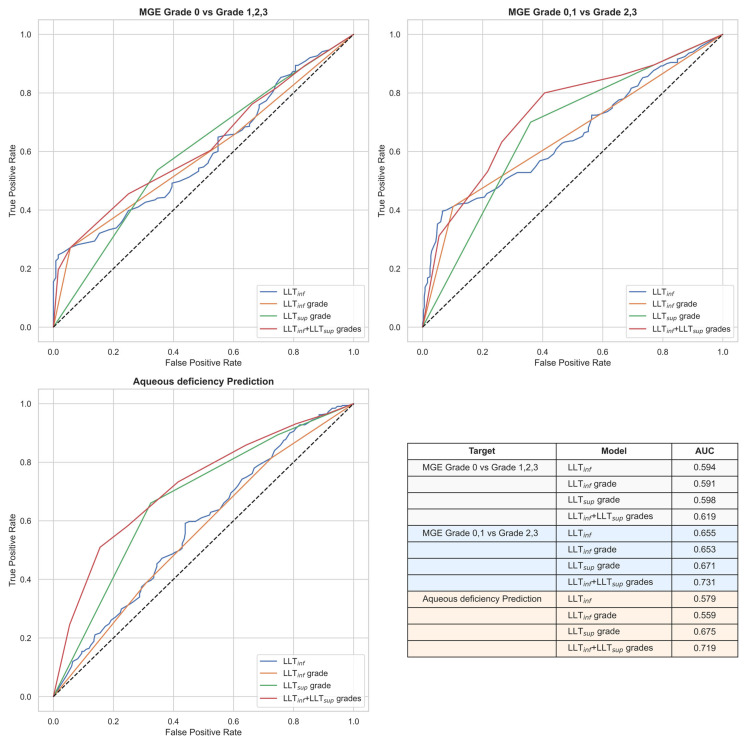
**Comparisons of the performance among inferior corneal lipid layer thickness (LLT*_inf_*), LLT*_inf_* grade, superior corneal lipid layer thickness (LLT*_sup_*) grade, and the combined LLT*_inf_* grade and LLT*_sup_* grade assessments in predicting meibomian gland expressibility (MGE, top) and aqueous deficiency (AD, bottom left).** Performance was evaluated using the area under the receiver operating characteristic curve (AUC) for the following three outcomes: (1) distinguishing MGE grade 0 from grades 1, 2, and 3 (top left), (2) distinguishing MGE grades 0 and 1 from grades 2 and 3 (top right), and (3) predicting aqueous deficiency (defined as TMH < 200 μm or Schirmer I ≤ 5 mm) (bottom left).

## 4. Discussion

This study demonstrates that LLT should not be interpreted solely as a marker of lipid secretion, but rather as a composite indicator influenced by both lipid expression and tear film distribution. In particular, increased inferior corneal LLT may represent lipid accumulation due to impaired distribution, especially in aqueous deficiency, rather than true LLT elevation in entire cornea. By integrating inferior and superior corneal LLT, this study provides a framework to distinguish between these conditions. These findings suggest that spatial assessment of LLT can improve the interpretation of tear film dynamics beyond conventional single-point measurements.

Superior corneal LLT provides additional context for this distinction: the increased gap between LLT*_inf_* and LLT*_sup_* suggests impaired lipid distribution, whereas high LLT*_sup_* indicates effective lipid spread. The absence of subjects in Group*_L–H_* further supports this model, as a thick superior lipid layer is unlikely without an adequate inferior lipid reservoir.

Previous studies have shown that DED indicators tend to be most favorable within a normal LLT range (approximately 60–100 nm), whereas reduced LLT is consistently associated with impaired meibomian gland function [14,26,27]. In contrast, the clinical significance of normal or increased LLT remains unclear. Although higher LLT has often been linked to reduced tear volume, findings have been inconsistent across studies. These discrepancies suggest that LLT reflects multiple underlying mechanisms rather than a single physiological process.

These discrepancies likely arise because LLT is influenced by other mechanisms in addition to meibum secretion. While reduced LLT reliably reflects impaired gland function, normal or increased LLT may result from either effective lipid expression or impaired distribution [5,14,26].

One explanation is impaired lipid distribution due to reduced aqueous volume, where limited upward spread leads to lipid accumulation in the inferior cornea [8,9]. Another hypothesis is compensatory lipid secretion in response to aqueous deficiency; however, supporting evidence remains limited [28]. The present findings support the distribution-based mechanism, particularly in eyes with high LLT*_inf_* and low LLT*_sup_*, where aqueous deficiency was more prevalent.

In addition to lipid distribution, the relationship between LLT and tear volume has also been proposed as a key factor contributing to these inconsistencies. Most studies have reported that higher LLT is associated with reduced tear volume [5,8,9,14,17,27], although this relationship has not been consistently observed [26]. Two main hypotheses have been suggested: one proposes compensatory aqueous secretion in response to reduced lipid delivery [28], whereas the other suggests that LLT increases under conditions of aqueous deficiency [8,9]. The findings of the present study support the coexistence of these mechanisms. In the low LLT*_inf_*, group, the proportion of aqueous deficiency was relatively lower than expected, suggesting a potential compensatory response. In contrast, higher LLT*_inf_* was associated with a greater prevalence of aqueous deficiency, particularly when LLT*_sup_* was low, supporting the hypothesis of impaired lipid distribution due to reduced aqueous volume. These findings indicate that LLT is modulated by both lipid–aqueous interactions and tear film dynamics, rather than reflecting a single underlying process.

SB appears to act as an important confounding factor in the interpretation of LLT-related tear film dynamics (see also Supplementary Materials S7). Consistent with previous reports, SB was associated with shorter TBUT but relatively preserved corneal staining [29]. Consistent with this, the present study demonstrated a similar discrepancy in the high LLT*_inf_* group, particularly in cases with high LLT*_sup_*, where SB was more prevalent despite relatively preserved corneal staining. This pattern may partially explain the apparent inconsistency between TBUT and ocular surface damage observed in eyes with increased LLT. Notably, SB was distributed across all LLT*_inf_* groups, suggesting that it is not directly related to a hypersecretory lipid state but may instead reflect other tear film abnormalities, such as mucin deficiency or inflammatory changes [4,30]. While these findings suggest a potential role of inflammatory mechanisms, direct inflammatory biomarkers were not assessed in this study, as the primary focus was on the functional interpretation of LLT in relation to tear film dynamics. Collectively, these findings indicate that such factors may act as confounders in LLT measurements, highlighting the need for further studies to clarify their contributions.

The performance of LLT-based DED subtype prediction was moderate, reflecting the multifactorial influences on LLT. Given the high prevalence of mixed-type DED and overlapping pathophysiology among subtypes, LLT is best regarded as an interpretive rather than standalone diagnostic tool [1,14]. However, these findings emphasize an integrative interpretation of tear film dynamics. LLT*_inf_* and LLT*_sup_* capture complementary aspects of tear film behavior—lipid accumulation and distribution—providing a more comprehensive framework than single-point measurements.

This study has several limitations. First, due to non-standardized distribution of variables across groups, nonparametric statistical tests were used, which limits the ability to interpret precise numerical differences between groups. However, this study did not aim to derive regression formulas or quantitative assessments but rather to establish a framework for evaluating tear film abnormalities. Second, LLT*_inf_* and LLT*_sup_* were assessed using different measurement approaches, with LLT*_inf_* providing quantitative values and LLT*_sup_* based on semi-quantitative grading. This difference in scale may introduce measurement variability and limit direct comparability. However, this dual-scale approach was intentionally adopted to capture complementary aspects of tear film dynamics, particularly lipid accumulation and distribution. Future development of quantitative methods for superior corneal LLT may further improve objectivity and clinical applicability. Third, the study population showed a predominance of female patients, reflecting the known higher prevalence of DED in women. Although the primary analyses were based on LLT-defined stratification, gender-related differences in tear film composition may have affected the results.

**Figure 3:**
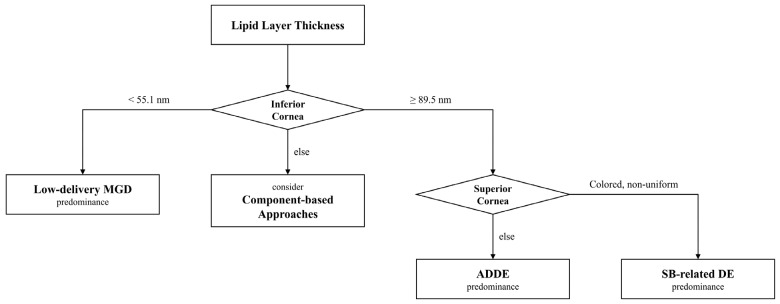
**Schematic framework for the clinical interpretation of tear interferometric lipid layer thickness (LLT) based on measurements at the inferior and superior cornea.** When the LLT of the inferior cornea (LLT*_inf_*) is <55.1 nm, low-delivery meibomian gland dysfunction (MGD) is primarily suspected. When LLT*_inf_* is between 55.1 and 89.5 nm, a component-based diagnostic approach is recommended to determine the underlying etiology. For LLT*_inf_* ≥ 89.5 nm, evaluation of the superior corneal LLT (LLT*_sup_*) may provide additional interpretive context: a colored and non-uniform LLT*_sup_* pattern may suggest tear film instability associated with spot break (SB), whereas a low LLT*_sup_* pattern may be associated with aqueous-deficient conditions. This framework is intended to support interpretation of LLT findings in the context of tear film dynamics rather than to serve as a definitive diagnostic algorithm.

However, clinically, this framework may be useful in two aspects. First, by assessing the TFLL distribution across the ocular surface, it offers a new perspective for evaluating ocular surface status with other dynamic or static DED assessments. Second, it may provide a potential indirect indicator of tear film component abnormalities or possibly ocular surface abnormalities such as altered mucin expression or ocular surface inflammation, which have not yet been widely integrated into clinical practice. These concepts are summarized in a schematic framework for clinical interpretation of LLT (Figure 3).

## 5. Conclusions

In conclusion, LLT reflects not only lipid secretion but also tear film distribution influenced by aqueous dynamics. Increased inferior corneal LLT does not necessarily indicate enhanced lipid expression, but may instead represent lipid accumulation due to impaired distribution. By integrating inferior and superior corneal LLT, this study provides a practical framework for distinguishing these mechanisms and improving the interpretation of LLT in DED.

Although the predictive performance of this approach remains moderate, combined spatial assessment of LLT may serve as a complementary tool for evaluating tear film dynamics and refining DED subtype interpretation. Future studies incorporating quantitative assessment of superior corneal LLT and integration with molecular or inflammatory biomarkers may further enhance the understanding of tear film dynamics and improve clinical applicability.

## Figures and Tables

**Table 1:** Characteristics of study subjects.

Characteristics	Units	Values
Age	years, Mean ± SD	49.5 ± 15.4
Sex	N (% of female)	478 (77.9%)
*Conventional DED assessments*		
OSDI	scores, Mean ± SD	28.7 ± 15.6
TBUT	seconds, Mean ± SD	3.5 ± 2.5
CSS	scores, Mean ± SD	0.5 ± 1.4
*Aqueous assessments*		
TMH	μm, Mean ± SD	207.6 ± 44.9
Schirmer I test	mm, Mean ± SD	6.7 ± 4.1
Aqueous deficiency	N (%)	318 (51.8%)
*Lipid assessments*		
MGE	grades, Mean ± SD	1.3 ± 0.9
Grade 0 (none)	N (%)	124 (20.2%)
Grade 1 (minimal to mild)	N (%)	240 (39.1%)
Grade 2 (moderate)	N (%)	176 (28.7%)
Grade 3 (severe)	N (%)	74 (12.0%)
MQ	grades, Mean ± SD	2.1 ± 0.9
Grade 0 (clear)	N (%)	22 (3.6%)
Grade 1 (cloudy)	N (%)	144 (23.5%)
Grade 2 (cloudy particulate)	N (%)	172 (28.0%)
Grade 3 (toothpaste or no secretion)	N (%)	276 (45.0%)
Lid margin abnormality		
Plugging	N (%)	135 (22.0%)
Telangiectasia	N (%)	117 (19.1%)
Anterior shift of MCJ	N (%)	202 (32.9%)
Notching	N (%)	120 (19.5%)
Epithelial desquamation	N (%)	157 (25.6%)
*Tear interferometric lipid layer thickness*		
LLT*_inf_*	nm, Mean ± SD	76.9 ± 26.5
Grade L (LLT*_inf_* < 55.1 nm)	N (%)	140 (22.8%)
Grade M (55.1 nm ≤ LLT*inf* < 89.5 nm)	N (%)	258 (42.0%)
Grade H (LLT*_inf_* ≥ 89.5 nm)	N (%)	216 (35.2%)
LLT*_sup_* grade		
Grade L (dark, uniform)	N (%)	330 (49.8%)
Grade M (gray, uniform and non-uniform)	N (%)	188 (32.4%)
Grade H (colored, non-uniform)	N (%)	96 (17.8%)
*Fluorescence tear break-up patterns*		
Dimple break	N (%)	85 (13.8%)
Spot break	N (%)	171 (27.9%)
Random break	N (%)	206 (33.6%)
Line break	N (%)	139 (22.6%)
Area break	N (%)	13 (2.1%)

Abbreviations: CSS, corneal staining score; DED, dry eye disease; LLT, lipid layer thickness; MCJ, mucocutaneous junction; MGE, meibomian gland expressibility; MQ, meibum quality; OSDI, ocular surface disease index; TBUT, fluorescence tear break-up time; TMH, tear meniscus height.

## Data Availability

The datasets presented in this article are not readily available because access is restricted in accordance with the conditions approved by the Institutional Review Board.

## Supplementary Materials

The following supporting information can be downloaded at: https://www.mdpi.com/article/10.3390/jcm15093553/s1.
