# Supplementary material for: Evaluating Dry Eye Disease Subtypes Based on Whole-Area Lipid Layer Thickness Assessment
Source: J Clin Med. 2026 May 6;15(9):3553. doi: 10.3390/jcm15093553 (PMC13163960; doi:10.3390/jcm15093553)
Supplement: Supplementary file 1 [file jcm-15-03553-s001.zip › jcm-4237908-supplementary.pdf]

SUPPLEMENTARY MATERIALS S1.

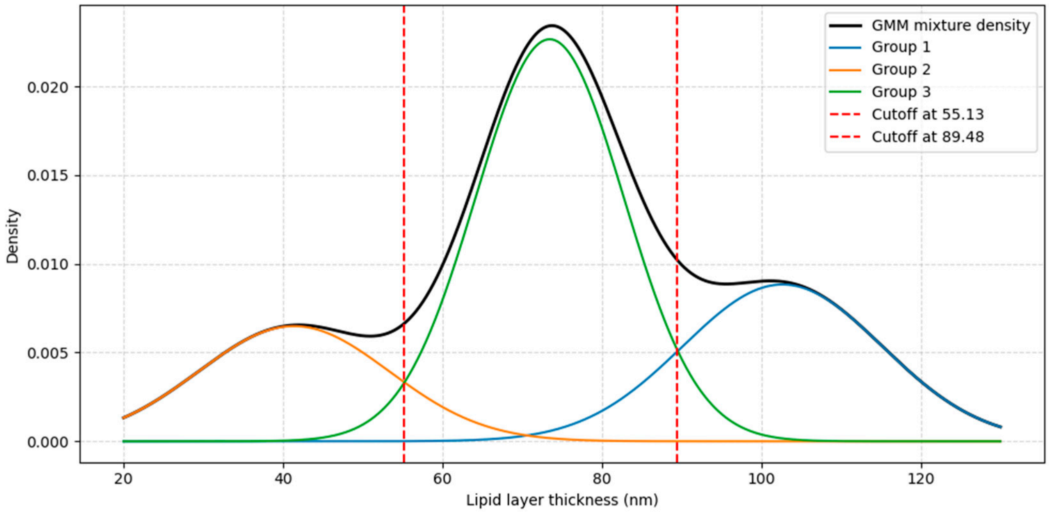

Supplementary Materials S1. To estimate objective cut-off values between three clinical subgroups, a Gaussian Mixture Model (GMM) was applied to datasets that replicate the observed group characteristics in the previous study.[15] Specifically, samples were analyzed from normal distributions using parameters corresponding to each group's sample size, mean, and standard deviation: Group 1 (n=54, mean=62.67, SD=21.19), Group 2 (n=11, mean=73.73, SD=12.33), and Group 3 (n=40, mean=89.58, SD=19.95). For each random seed from 0 to 99, the data were evaluated for conformity to the reference distribution, defined as within ±5% of the target mean and standard deviation. Only seeds satisfying this criterion for all three groups were retained. For these qualified cases, a GMM with three components was fitted using the Expectation-Maximization algorithm, and the two cut-off points were identified at the intersection of adjacent component responsibilities. Mean and standard deviation of the estimated cut-offs were reported based on all qualified repetitions. This approach reflects both distributional fidelity and model-based uncertainty.

SUPPLEMENTARY MATERIALS S2.

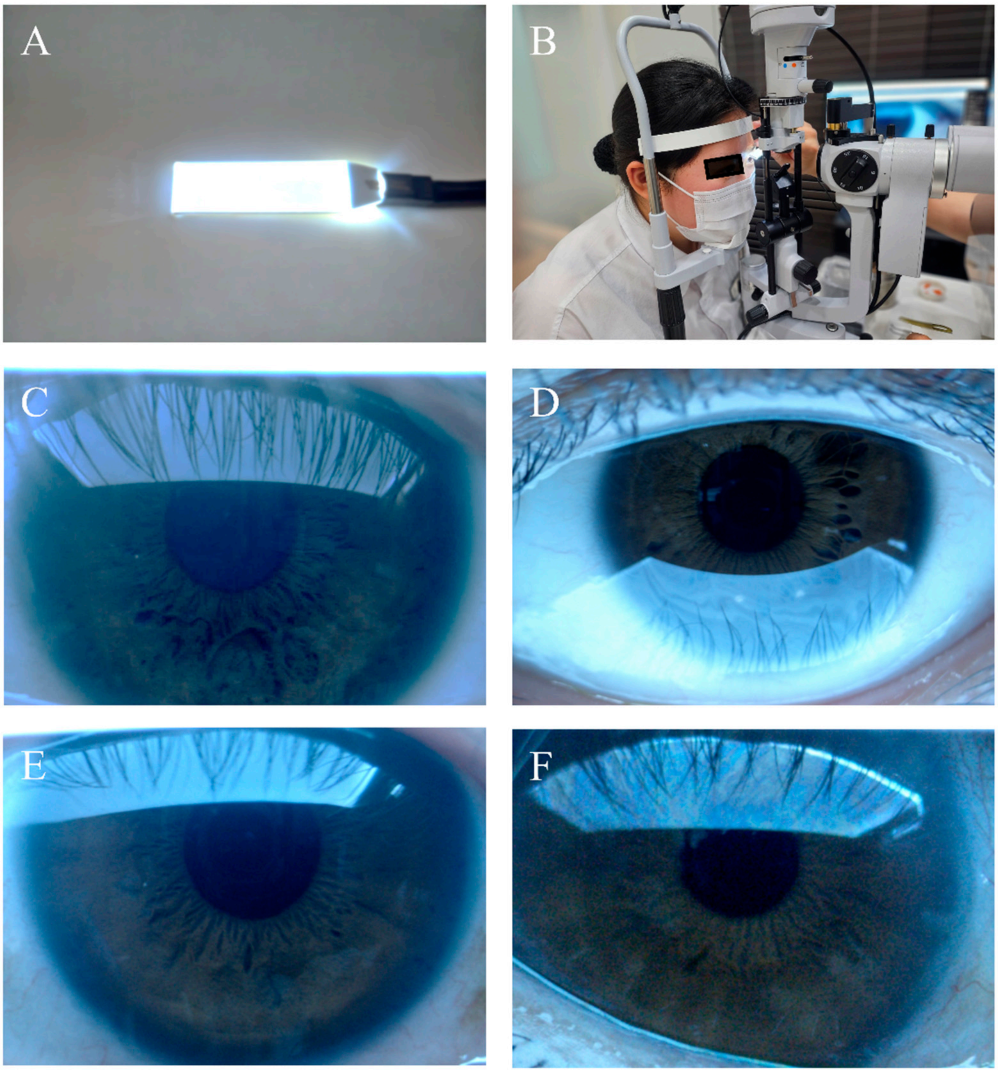

Supplementary Materials S2. Device (A, B) and examples (C, D, E, F) of manual lipid layer thickness (LLT) measurement. (A) Whitish LED plate used for measuring superior corneal LLT in this study. (B) Measurement performed under slit-lamp examination, followed by anterior segment photography. The illumination pattern of the superior cornea was classified into three grades using a modified version of a previously established system.[14] (C) A dark, uniform pattern, classified as Level 0. (D) A gray, non-uniform pattern, classified as Level 1. (E) A gray, uniform pattern, classified as Level 1. (F) A colored, non-uniform pattern, classified as level 2.

SUPPLEMENTARY MATERIALS S3.

Supplementary Materials S3. The intra-/interclass correlation coefficient of the superior corneal lipid layer thickness (LLTsup) and fluorescein tear break-up pattern (FTBUP) measurements

**LLT*sup***	Fleiss κ	95% confidence interval	
		Lower	Upper
Grade L (dark, uniform)	0.731	0.525	0.938
Grade M (gray, uniform and non-uniform)	0.679	0.472	0.885
Grade H (colored, non-uniform)	0.949	0.743	1.156
Total	0.791	0.642	0.941

**FTBUP**	Fleiss κ	95% confidence interval	
		Lower	Upper
Dimple break	0.520	0.313	0.727
Spot break	1.000	0.793	1.207
Random break	0.750	0.430	0.844
Line break	0.850	0.601	1.014
Area break	1.000	0.793	1.207
Total	0.772	0.666	0.879

Three independent examiners assessed manual tear interferometry images and fluorescein tear break-up videoclips. All 310 study subjects were evaluated, and the most favored classification was used in the study.

SUPPLEMENTARY MATERIALS S4.

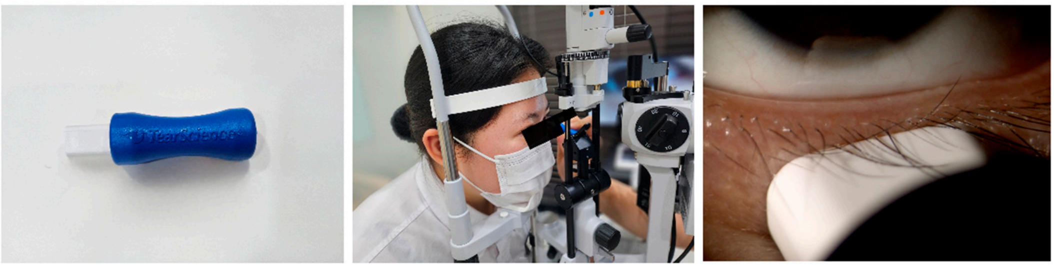

Supplementary Materials S4. The Meibomian Gland Evaluator (Left, Johnson & Johnson Vision, Jacksonville, Florida, United States). Meibomian gland expression was measured at a pressure of 0.3 pounds per square inch (approximately 15 mmHg) using the Meibomian Gland Evaluator in the central 8 glands of the upper and lower eyelids.

SUPPLEMENTARY MATERIALS S5.

Supplementary Materials S5. Names and number of lipid layer thickness (LLT) groups

	LLT group	LLT_{sup} Grade L (Dark, uniform)	Grade M (Gray, uniform and non-uniform)	Grade H (Colored, non-uniform)
LLT_{inf}	Grade L (LLT < 55.1 nm)	Group $_{L\text{-}L}$ N = 98	Group $_{L\text{-}M}$ N = 42	Group $_{L\text{-}H}$ N = 0
	Grade M (55.1 nm ≤ LLT < 89.5 nm)	Group $_{M\text{-}L}$ N = 114	Group $_{M\text{-}M}$ N = 107	Group $_{M\text{-}H}$ N = 37
	Grade H (LLT ≥ 89.5 nm)	Group $_{H\text{-}L}$ N = 94	Group $_{H\text{-}M}$ N = 50	Group $_{H\text{-}H}$ N = 72

SUPPLEMENTARY MATERIALS S6.

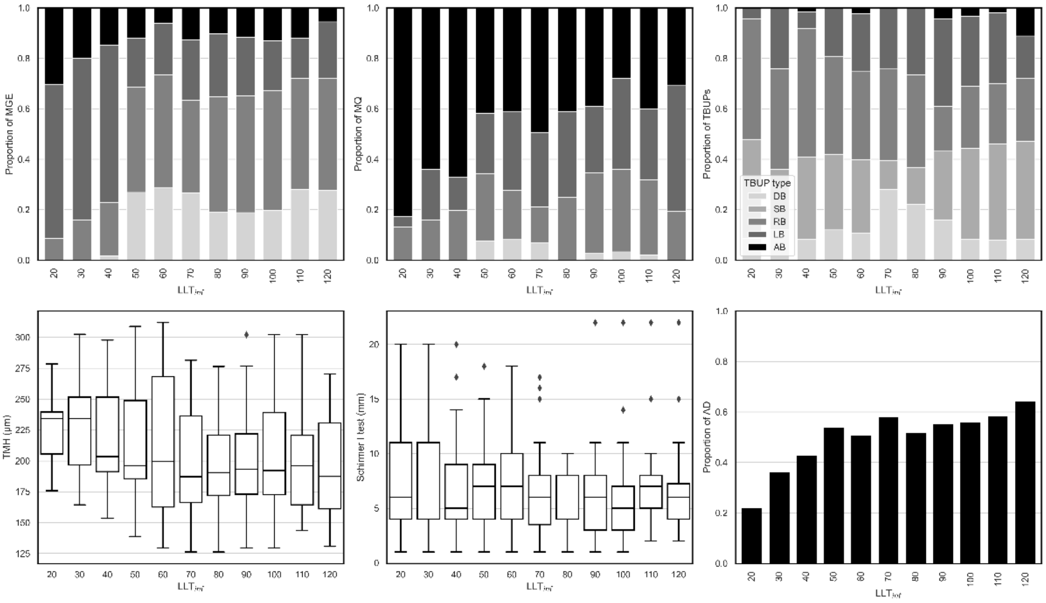

Supplementary Materials S6. Meibomian gland functionality (top left and middle), tear break-up patterns (TBUPs, top right), and aqueous volume (bottom) of inferior corneal lipid layer thickness (LLT_{inf}). Meibomian gland functionality was evaluated using meibomian gland expressibility (MGE) and meibum quality (MQ). Aqueous volume was assessed based on tear meniscus height (TMH, bottom left) measured by Keratograph 5M® and Schirmer I test (bottom middle). Aqueous deficiency (AD, bottom right) was defined as either TMH < 200 μm or Schirmer I ≤ 5 mm.

SUPPLEMENTARY MATERIALS S7.

Supplementary Materials S7. Comparison of Dry Eye Parameters According to Inferior and Superior Corneal LLT (LLT_{inf} and LLT_{sup}) Grades

	LLT_{inf} Grade	Value	*p*-value	LLT_{sup} Grade	Value	*p*-value
OSDI	Low	29.6 ± 10.1	0.332 (vs. M)	Low	30.0 ± 10.0	0.652 (vs. M)
(scores)				Middle	28.7 ± 9.6	
	Middle	27.4 ± 13.0	0.385 (vs. H)	Low	30.3 ± 12.6	<0.001 (vs. M)*
				Middle	24.0 ± 10.5	0.712 (vs. H)
				High	26.3 ± 10.0	0.138 (vs. L)
	High	29.6 ± 14.2	1.000 (vs. L)	Low	32.2 ± 9.8	0.124 (vs. M)
				Middle	28.1 ± 13.0	0.368 (vs. H)
				High	26.3 ± 11.5	0.010 (vs. L)*
TBUT	Low	3.4 ± 1.6	0.028 (vs. M)*	Low	3.4 ± 1.5	0.290 (vs. M)
(seconds)				Middle	2.3 ± 1.4	
	Middle	3.9 ± 2.3	<0.001 (vs. H)*	Low	3.8 ± 2.0	0.721 (vs. M)
				Middle	4.0 ± 2.3	0.256 (vs. H)
				High	3.6 ± 1.8	0.685 (vs. L)
	High	3.0 ± 2.0	0.150 (vs. L)	Low	3.4 ± 1.6	0.443 (vs. M)
				Middle	3.1 ± 1.4	0.030 (vs. H)*
				High	2.4 ± 1.4	<0.001 (vs. L)*
CSS	Low	0.3 ± 1.1	1.000 (vs. M)	Low	0.3 ± 1.0	0.985 (vs. M)
(scores)				Middle	0.3 ± 1.2	
	Middle	0.4 ± 1.1	0.016 (vs. H)*	Low	0.6 ± 1.0	0.013 (vs. M)*
				Middle	0.2 ± 0.6	0.158 (vs. H)
				High	0.4 ± 0.6	0.442 (vs. L)
	High	0.8 ± 1.2	0.002 (vs. L)*	Low	1.1 ± 1.3	0.017 (vs. M)*
				Middle	0.6 ± 0.6	0.077 (vs. H)
				High	0.4 ± 0.4	<0.001 (vs. L)*
MGE	Low	1.9 ± 0.8	<0.001 (vs. M)*	Low	2.0 ± 0.7	0.013 (vs. M)*
(grades)				Middle	1.6 ± 0.9	
	Middle	1.1 ± 0.9	1.000 (vs. H)	Low	1.4 ± 0.9	<0.001 (vs. M)*
				Middle	0.9 ± 0.8	0.915 (vs. H)
				High	1.0 ± 0.8	0.036 (vs. L)*
	High	1.2 ± 0.9	<0.001 (vs. L)*	Low	1.4 ± 0.9	0.060 (vs. M)
				Middle	1.0 ± 0.9	1.000 (vs. H)
				High	1.1 ± 1.0	0.115 (vs. L)
MQ	Low	2.5 ± 0.8	<0.001 (vs. M)*	Low	2.5 ± 0.9	0.887 (vs. M)
(grades)				Middle	2.5 ± 0.5	
	Middle	2.1 ± 0.9	1.000 (vs. H)	Low	2.3 ± 0.8	0.004 (vs. M)*
				Middle	1.9 ± 1.0	1.000 (vs. H)
				High	1.9 ± 1.0	0.027 (vs. L)*
	High	2.0 ± 0.9	<0.001 (vs. L)*	Low	2.1 ± 0.9	0.534 (vs. M)
				Middle	2.0 ± 0.8	1.000 (vs. H)
				High	1.9 ± 0.8	0.165 (vs. L)
TMH	Low	219.7 ± 39.3	0.005 (vs. M)*	Low	211.3 ± 36.7	<0.001 (vs. M) *
(μm)				Middle	239.3 ± 38.6	
	Middle	207.0 ± 48.9	1.000 (vs. H)	Low	188.6 ± 44.2	<0.001 (vs. M)*
				Middle	220.2 ± 49.5	0.549 (vs. H)
				High	225.6 ± 42.0	<0.001 (vs. L)*
	High	200.4 ± 41.8	0.001 (vs. L)*	Low	178.1 ± 26.0	0.001 (vs. M)*
				Middle	201.3 ± 39.7	0.002 (vs. H)*
				High	228.9 ± 42.9	<0.001 (vs. L)*
Schirmer	Low	7.3 ± 4.9	0.467 (vs. M)	Low	5.6 ± 4.8	0.001 (vs. M)*
(mm)				Middle	8.0 ± 4.5	
	Middle	6.7 ± 3.9	0.373 (vs. H)	Low	5.9 ± 4.0	0.001 (vs. M)*
				Middle	7.3 ± 3.7	1.000 (vs. H)
				High	7.4 ± 3.9	0.040 (vs. L)*
	High	6.3 ± 3.6	0.231 (vs. L)	Low	4.9 ± 2.1	0.151 (vs. M)
				Middle	5.6 ± 2.1	<0.001 (vs. H)*
				High	8.6 ± 4.6	<0.001 (vs. L)*
AD	Low	42.1	0.158 (vs. M)	Low	48.9	0.013 (vs. M)*
(%)				Middle	26.2	
	Middle	52.3	0.807 (vs. H)	Low	73.7	<0.001 (vs. M)*
				Middle	37.4	1.000 (vs. H)
				High	29.7	<0.001 (vs. L)*

	High	57.4	0.015 (vs. L)*	Low	83.0	<0.001 (vs. M)*
				Middle	46.0	0.350 (vs. H)
				High	31.9	<0.001 (vs. L)*
TBUPs† (%)	Low	8.6/30.7/47.9/12.1/0.7	<0.001 (vs. M)*	Low	7.1/27.6/51.0/13.3/1.0	0.386
				Middle	11.9/38.1/40.5/9.5/0.0	
	Middle	19.4/20.9/35.3/23.6/0.8	<0.001 (vs. H)*	Low	8.8/21.1/32.5/36.8/0.8	<0.001 (vs. M)*
				Middle	29.9/18.6/37.4/13.1/1.0	<0.001 (vs. H)*
				High	21.6/27.1/37.8/13.5/0.0	<0.001 (vs. L)*
	High	10.6/34.3/23.2/28.3/4.6	<0.001 (vs. L)*	Low	11.7/23.4/16.0/40.4/8.5	<0.001 (vs. M)*
				Middle	12.0/30.0/34.0/20.0/4.0	<0.001 (vs. H)*
				High	8.3/51.4/25.2/18.1/0.0	<0.001 (vs. L)*

Abbreviation: AD, aqueous deficiency; CSS, corneal staining score; LLT, lipid layer thickness; MGD, meibomian gland dysfunction; MGE, meibomian gland expressibility; MQ, meibum quality; OSDI, ocular surface disease index; TBUP, tear break-up pattern; TBUT, tear break-up time; TMH, tear meniscus height.

* p <0.05, p-value was calculated with Kruskal-Wallis test for continuous variables, Jonckheere-Terpstra test for ranked variables, and chi-square test for nominal variables. Bonferroni's test was conducted as a post-hoc analysis following Kruskal-Wallis test.

† Tear break-up patterns are listed in the order of dimple break (DB), spot break (SB), random break (RB), line break (LB), and area break (AB). Each value represents the percentage of cases exhibiting each pattern.

SUPPLEMENTARY MATERIALS S8.

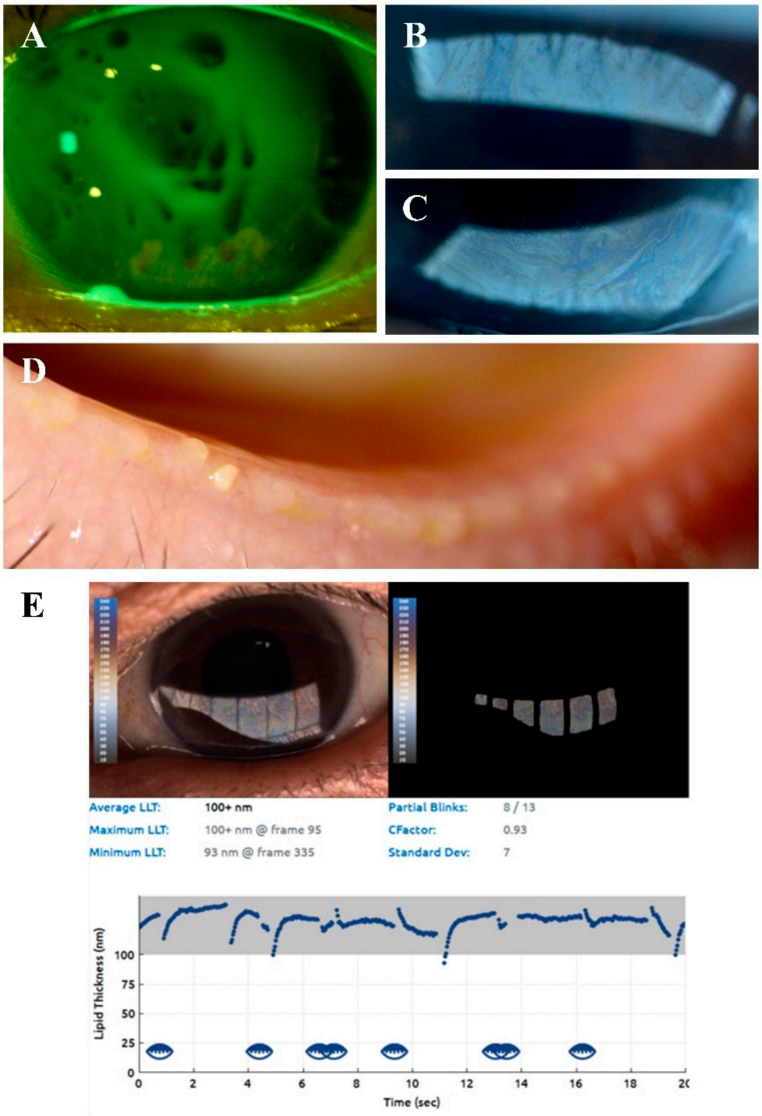

Supplementary Materials S8. A case example of 66-year-old female patient. The left eye of the patient presented with a tear meniscus height of 209 μm, and no signs of corneal erosion were observed. The fluorescein tear break-up pattern indicated a "spot break" type with a tear break-up time of under 1 second, occurring immediately after the up-motion of the upper eyelid (A). Both the inferior and superior cornea showed a high lipid layer thickness (LLT) with a colored, non-uniform pattern (B, C). After eyelid squeezing, meibomian gland expressibility was graded as 0, and meibum quality was graded as 1 (D). The inferior LLT, measured using the LipiView® II Ocular Surface Interferometer, was 112 nm (E).
